# Platinum resistant recurrence and early recurrence in a multi-centre cohort of patients undergoing interval cytoreductive surgery for advanced epithelial ovarian cancer

**DOI:** 10.3389/fonc.2022.951419

**Published:** 2022-09-02

**Authors:** Aditi Bhatt, Snita Sinukumar, Vahan Kepenekian, Praveen Kammar, Sanket Mehta, Sakina Shaikh, Witold Gertych, Naoual Bakrin, Olivier Glehen

**Affiliations:** ^1^ Department of Surgical Oncology, Zydus Hospital, Ahmedabad, India; ^2^ Department of Surgical Oncology, Jehangir Hospital, Pune, India; ^3^ Department of Surgical Oncology, Centre Hospitalier Lyon-sud, Lyon, France; ^4^ Department of Surgical Oncology, Saifee Hospital, Mumbai, India; ^5^ Department of Gynecology, Centre Hospitalier Lyon-sud, Lyon, France

**Keywords:** advanced ovarian cancer, interval cytoreductive surgery, Hyperthermic intraperitoneal chemotherapy (HIPEC), total parietal peritonectomy, early recurrence, platinum resistant recurrence

## Abstract

**Background:**

Aggressive locoregional therapies like hyperthemic intraperitoneal chemotherapy(HIPEC) and total parietal peritonectomy(TPP) have been used to delay recurrence in patients with advanced ovarian cancer undergoing interval cytoreductive surgery(CRS). The aim of this retrospective study was to evaluate the incidence of platinum resistant recurrence (PRR) and early recurrence (ER)(recurrence within 6 months and 1 year of the last dose of platinum based therapy, respectively) in patients undergoing interval CRS. The secondary goal was to study impact of each of these therapies on PRR and ER.

**Methods:**

One-hundred and fifty-three patients undergoing interval CRS from July 2018 to June 2020 were included. The surgical strategy was to perform a TPP in which the entire parietal peritoneum is resected irrespective of the disease extent or a selective parietal peritonectomy (SPP) in which only the peritoneum bearing visible residual disease is resected. The use of HIPEC was at the discretion of the treating oncologists.

**Results:**

The median surgical PCI was 15 [range, 0-37]. A CC-0 resection was obtained in 119 (77.7%) and CC-1 in 29 (18.9%) patients. Eighty-one (53%) patients had a TPP and 72 (47%) had SPP. HIPEC was performed in 98(64%) patients. Bevacizumab maintenance was administered to 31(19.6%) patients. No patients received PARP inhibitors during first-line therapy. PRR was observed in 8(5.2%) patients and ER in 30(19.6%). The respective incidences of PRR and ER were 4.9% and 16% in the TPP group, 4.1% and 23.6% in the SPP group, 9% and 20% in the no-HIPEC group and 3% and 19.3% in the HIPEC groups. On multivariate analysis, CC-0(p=0.014) resection and HIPEC(p=0.030) were independent predictors of a low ER. All patients with PR and 70% with ER had peritoneal recurrence with or without extra-peritoneal sites of recurrence.

**Conclusions:**

The incidence of PRR and ER in this cohort was low as compared to historical data. This low incidence could be attributed to the use of aggressive locoregional therapies like TPP and HIPEC. In future, studies should be conducted to confirm these findings and evaluate the potential additive benefit of TPP and HIPEC coupled together as well as their combination with maintenance therapies.

## Introduction

Advanced ovarian cancer remains an incurable disease despite the advances in surgical strategies and systemic therapies. In stages III-C and IV-A that are treated with a combination of cytoreductive surgery(CRS) and systemic chemotherapy, the sequencing of these treatments has been a topic of debate and research for the past couple of decades (1). Nevertheless, many patients who present with advanced unresectable disease are treated with neoadjuvant chemotherapy(NACT) followed by interval CRS. The reported incidence of platinum resistant recurrence(PRR) is higher in patients undergoing NACT compared to those undergoing primary CRS (2).

The complete resection of macroscopic disease (CC-0 resection) or a complete gross resection (CGR) is one of the most important prognostic factors in ovarian cancer (3). In case of interval CRS, the conventional strategy is to resect only sites of residual macroscopic disease. Some researchers suggested that such a strategy could be insufficient since areas that have responded to NACT may harbor occult disease that has a high likelihood of harboring chemotherapy resistant cells and could increase the risk of recurrence (4, 5). The proposed alternative strategy is to systematically resect the entire parietal peritoneum(total parietal peritonectomy-TPP), that is invariably involved prior to NACT in patients presenting with unresectable disease (6). Though there is no robust evidence demonstrating the benefit of such extensive surgery, early reports show that the morbidity of TPP is acceptable and the incidence (40%) of occult disease in high (7–9). The distribution of residual disease in the peritoneal cavity (significantly higher incidence of both occult and overt disease in the parietal peritoneum compared to the visceral peritoneum) favors this approach (7–9).

The OVHIPEC-1 trial demonstrated the benefit of adding hyperthermic intraperitoneal chemotherapy (HIPEC) to interval CRS (10). The underlying mechanism is probably the ability of HIPEC to address microscopic residual disease more effectively and prevent implantation of free intraperitoneal cancer cells shed during surgery.

Maintenance therapy with the anti-VEGF agent bevacizumab has shown a significantly longer progression-free survival in patients with advanced ovarian cancer, with a benefit in overall survival mainly in patients with suboptimal surgery and stage IV disease (11, 12). For patients with BRCA mutations and mismatch-repair deficiency, the use of Poly ADP-Ribosyl Polymerase(PARP) inhibitors has been associated with a significant benefit in the progression-free (PFS) but overall-survival (OS) results are awaited (13, 14). The role of such maintenance therapies in patients undergoing aggressive locoregional therapies like HIPEC and TPP has not been evaluated.

In this study, our goal was to evaluate the incidence of platinum resistant recurrence and early recurrence (recurrence within 6 months and 1 year of the last dose of platinum based therapy, respectively) in a multi-center cohort of patients undergoing interval CRS. The secondary aim was to study the impact of various prognostic factors including the type of peritonectomy and HIPEC on PRR and early recurrence (ER).

## Methods

This is a retrospective analysis of prospectively collected data. Four centers contributed to this study: three from India and one from France. Ethical approval was obtained at all four participating centers (Institutional review board (IRB) no A15-128 for Hospital Lyon-Sud; specific IRB numbers are not allotted at the three Indian centers). Written informed consent was obtained from all patients. Patients with advanced epithelial ovarian, fallopian tube and primary peritoneal cancer (stage IIIC) undergoing interval CRS following NACT were included in the study. Patients undergoing upfront CRS, second look surgery or those who did not undergo surgery after NACT were excluded. At all centers, patients in whom a CC-0 resection was not deemed possible after the initial work-up that included a staging laparoscopy were treated with NACT. Interval CRS was performed after 3-6 cycles NACT. Imaging comprised of one or more of the following – CT scan, MRI and PET CT and was performed within 15 days of the planned surgical procedure. A re-staging laparoscopy was performed at the discretion of the operating surgeon.

### Surgical intervention

All surgical procedures were performed with the goal of obtaining a complete cytoreduction (no visible residual disease). Briefly, a midline incision from the xiphoid to the pubis was employed irrespective of the disease extent. The disease was quantified using Sugarbaker’s peritoneal cancer index (PCI) (15). For all patients, the falciform and the umbilical round ligament were systematically resected and visceral resections were performed for organs involved by tumor (16). There were two surgical strategies for addressing the peritoneal disease. At the French center, a selective parietal peritonectomy (SPP) comprising resection of disease bearing areas of the peritoneum and a systematic supracolic omentectomy were performed. At the three Indian centers, a total parietal peritonectomy (TPP) was systematically performed, irrespective of the disease extent, as part of a registered protocol (CTRI 2018/12/016789) (7). TPP comprised the following peritonectomies: pelvic, antero-parietal, right and left upper quadrant together with a total omentectomy (greater and lesser omentectomy). A total mesenteric peritonectomy was not part for that protocol.

The completeness of cytoreduction was reported using the completeness of cytoreduction score (CC-score) (15). A bilateral pelvic and retroperitoneal lymphadenectomy was performed in case of suspicious lymph nodes on imaging or intraoperatively, as per the recommendations after the LION trial.

### HIPEC

At the French centre, HIPEC was performed using the OVIHIPEC-1 protocol (Cisplatin 100mg/m² for 90min, combined with intravenous Sodium Thiosulfate), by the closed method, unless there was a contraindication to the procedure (10). HIPEC is an out-of-pocket expenditure for patients in India and was performed only for those who could afford that additional cost and consented for the procedure. HIPEC was performed with cisplatin 75mg/m^2^ for 90 minutes by the open (2 centres) or closed method (1 centre). The dose of 100mg/m2 was not used due to the non-availability of sodium thiosulfate (10).

### Evaluation of morbidity

The 90-day morbidity and mortality were recorded. The common toxicology criteria for adverse events (CTCAE) version 4.3 classification was used to record the morbidity (17). Grades 3 and 4 were considered major morbidity.

### Pathological evaluation

The pathological evaluation was performed using a previously defined protocol for peritonectomy specimens and based on the existing guidelines for the ovarian primary and regional nodes (18, 19). Appropriate immunohistochemistry markers were used to confirm the presence of disease when required. The PeRitOneal MalIgnancy Stage Evaluation online application (e-PROMISE) was used to define anatomical structures in each region of the peritoneal cancer index (20). The peritoneal cavity was divided into 4 regions: the upper region comprising regions 1,2,3, middle region comprising regions 0, 4, 8, the lower region comprising regions 5,6,7 and the small bowel regions (9-12).

The pathological PCI was calculated on the lines of the surgical PCI (21). The retroperitoneal nodes and those dissected with the resected segments of bowel and omentum were analyzed.

The pathological response to chemotherapy was graded based on the chemotherapy response score developed by Bohm et al. (22).

BRCA mutation testing was performed for all patients at the French centre and for selected patients at the Indian centres.

### Adjuvant chemotherapy and maintenance therapies

Adjuvant chemotherapy was started within 4-6 weeks of surgery and continued up to 6 cycles. For patients receiving all 6 cycles before surgery, an additional 2 to 3 cycles were administered at the discretion of the treating oncologist. Maintenance therapy with bevacizumab was also at the discretion of the oncologist.

### Follow-up

Routine 3-monthly follow-up included clinical exam, CA-125 dosage and cross-sectional imaging studies as deemed suitable for the first two years and 6-monthly thereafter. The diagnosis of recurrence was made according to the Gynecologic Cancer Inter Group (GCIC) criteria (23). Recurrence within 6 months (platinum resistant recurrence) and within 12 months(early recurrence) of completion of the last dose of platinum-based chemotherapy was recorded.

### Statistical analysis

Categorical data were described as number (%). Abnormally distributed continuous data were expressed as the median and range. Categorical data were compared with the x2 test. For comparison of means, the independent sample t test was used and for medians, the Mann-Whitney U test was used. A p-value of <0.05 was considered statistically significant. The impact of various prognostic factors on recurrence within 12 months was evaluated using logistic regression analysis. This analysis was only performed on patients who had completed 12 months of follow-up. The prognostic factors that were evaluated were the surgical and pathological PCI, number of NACT cycles, CC-score, HIPEC, lymph node involvement, extent of peritoneal resection (TPP or SPP), chemotherapy response grade (the term is used instead of chemotherapy response score to avoid confusion with CRS), grade 3-4 complications rates and the use of maintenance bevacizumab.

## Results

From July 2018 to June 2020, 153 patients undergoing interval CRS with or without HIPEC and having a minimum follow-up of 6 months from the last dose of platinum based chemotherapy were included. All patients had serous carcinoma of the ovary, fallopian tube or that arising from the peritoneum. 101 (66%) patients received 3-4 cycles of NACT and 52 (34%) received more than 4 cycles. The median surgical PCI was 15 [range, 0-37]. A CC-0 resection was obtained in 119 (77.7%) and CC-1 in 29 (18.9%) patients.

HIPEC was performed for 98 (64%) patients (**Table 1**). 81 (53%) patients had a TPP and 72 (47%) had SPP **(** **Table 2** **)**. The 90-day major morbidity was 29.4% (45 patients) and 3 (1.9%) patients died within 90 days of surgery. The details of the complications and a comparison between the HIPEC and non-HIPEC groups are provided in **Table 3**. Adjuvant chemotherapy was started within 6 weeks for the 147 (96%) patients who received it and 145(94.7%) patients completed the stipulated adjuvant chemotherapy. Bevacizumab maintenance was administered to 31(19.6%) patients. BRCA 1 or 2 mutations were seen in 10/80 (12.5%) patients. No patients received PARP inhibitors.

**Table 1:** Comparison between patients treated with or without HIPEC.

Clinical parameter		All patients n = 153 (%)	No HIPEC N = 55 (%)	HIPEC N = 98 (%)	p-value
Age	<50 >50	28 (18.3)	12 (21.8)	16 (16.3)	0.399
		125 (81.7)	43 (78.2)	82 (83.7)	
Number of NACT cycles	3-4	101 (66.0)	37 (67.2)	64 (65.3)	0.805
	>4	52 (34.0)	18 (32.8)	34 (34.7)	
Surgical PCI	0-9	37 (24.1)	17 (30.5)	20 (20.4)	0.400
	10-19	77 (50.3)	26 (47.2)	51 (52.0)	
	20-29	36 (23.5)	12 (21.8)	24 (41.3)	
	30-39	3 (1.9)	2 (3.6)	1 (1.7)	
Median surgical PCI		15 [0-37]	13 [0-37]	15 [3-30]	0.540
CC-score	CC-0	119 (77.7)	42 (76.3)	77 (78.5)	0.007
	CC-1	29 (18.9)	8 (14.5)	21 (21.4)	
	CC-2/3	5 (3.2)	5 (9.0)	0 (0.0)	
Peritonectomy approach	SPP	72 (47.0)	8 (14.5)	64 (65.3)	<0.001
	TPP	81 (53.0)	47 (85.5)	34 (34.7)	
Number of peritonectomies	0-6 7	54 (35.2) 99 (64.8)	8 (14.5) 47 (85.5)	46 (46.9) 52 (53.1)	<0.001
Organ resections	0-3 4 -5 >5	71(46.4) 59 (38.5) 23 (15.0)	23 (41.8) 23 (41.8) 9 (16.3)	48 (48.9) 36 (36.7) 14 (14.2)	0.695
Grade 3-4 complications		45 (29.4)	9 (16.3)	36 (36.7)	0.002
90-day mortality		3 (1.9)	0 (0.0)	3 (3.0)	0.643
Pathological PCI	0-9	85 (55.5)	30 (54.5)	55 (56.1)	0.922
	10-19	61 (39.8)	22 (40.0)	39 (39.7)	
	20-29	7 (4.5)	3 (5.4)	4 (4.0)	
	30-39	0 (0.0)	0 (0.0)	0 (0.0)	
Median pathological PCI		8 [0-26]	9 [0-26]	8 [0-26]	0.430
Involvement of upper regions		94 (61.4)	33 (60.0)	61 (0.0)	0.784
Small bowel involvement		55 (35.9)	17 (30.9)	38 (38.7)	0.330
Chemotherapy response score	CRG	4 (2.6) 23 (15.0) 126 (82.3)	1 (1.8)	3 (3.0)	0.887
			8 (14.5)	15 (15.3)	
			46 (83.6)	80 (81.6)	
Regional lymph node involvement		46 (30.0)	14 (25.4)	32 (32.6)	0.351
BRCA 1 or 2 mutations*		10 (6.5)*	1(1.8)	9 (9.1)	0.076
Bevacizumab		31 (19.6)	2 (1.8)	29 (29.5)	<0.001
Recurrence within 6 months of surgery		8 (5.2)	5 (9.0)	3 (3.0)	0.107
Recurrence in 6-12 months		22 (14.3)	6 (10.0)	16 (16.3)	0.359
Recurrence in 0-12 months		30 (19.6)	11 (20.0)	19 (19.3)	0.972

*10/80 (12.5%) patients in whom BRCA testing was done.

**Table 2:** Comparison of patients treated with TPP and SPP^.

Clinical parameter		All patients n = 153 (%)	TPP N = 81 (%)	SPP N = 72 (%)	p-value
Age	<50 >50	28 (18.3) 125 (81.7)	21 (25.9) 60 (74.1)	7 (9.7) 65 (90.3)	0.009
Number of NACT cycles	3-4 >4	101 (66.0) 52 (34.0)	59(72.8) 22 (27.2)	42 (58.3) 30 (41.7)	0.058
Surgical PCI	0 - 9 10 - 19 20 - 29 30 - 39	37 (24.1) 77 (50.3) 36 (23.5) 3 (1.9)	15 (18.5) 38 (46.9) 25 (30.8) 3 (3.7)	22 (30.5) 39 (54.1) 11 (15.2) 0 (0.0)	0.060
Median surgical PCI		15 [0-37]	15 [0-37]	13 [0-28]	0.131
CC - score	CC-0 CC - 1 CC - 2/3	119 (77.7) 29 (18.9) 5 (3.2)	58 (71.6) 18 (22.2) 5 (6.1)	61 (84.8) 11 (15.2) 0 (0.0)	0.133
HIPEC		98	34 (41.9)	64 (88.8)	<0.001
Number of peritonectomies	0 - 6 7	54 (35.2) 99 (64.8)	0 (0.0) 81 (100.0)	54 (75.0) 18 (25.0)	<0.001
Organ resections	0 - 3 4 - 5 >5	71(46.4) 59 (38.5) 23 (15.0)	29 (35.8) 34 (41.9) 18 (22.2)	42 (58.3) 25 (34.7) 5 (6.9)	0.004
Grade 3-4 complications		45 (29.4)	15 (18.5)	30 (41.6)	0.001
90-day mortality		3 (1.9)	3 (3.7)	0 (0.0)	0.363
Pathological PCI	85 61 7 0	85 (55.5) 61 (39.8) 7 (5.5) 0 (0.0)	39 (48.1) 38 (46.9) 4 (4.9) 0 (0.0)	46 (63.8) 23 (31.9) 3 (4.1) 0 (0.0)	0.142
Median pathological PCI		8 [0-26]	10 [0-26]	7 [0-21]	0.080
Involvement of upper regions		94 (61.4)	52 (64.1)	42 (58.3)	0.456
Small bowel involvement		55 (35.9)	27 (33.3)	28 (38.8)	0.474
Chemotherapy response score		4 (2.6) 23 (15.0) 126 (82.3)	2 (2.4) 13 (16.0) 66 (81.4)	2 (2.7) 10 (13.8) 60 (83.3)	0.928
Regional lymph node involvement		46 (30.0)	27 (33.3)	19 (26.3)	0.349
BRCA 1 or 2 mutations		10 (6.5)*	2 (2.4)	8 (11.1)	0.998
Bevacizumab		31 (19.6)	0 (0.0)	31 (43.0)	<0.001
Recurrence in 0-6 months of surgery		8 (5.2)	5 (4.9)	3 (4.1)	0.577
Recurrence in 6-12 months		22 (14.3)	8 (6.1)	14 (19.4)	0.092
Recurrence in 0-12 months		30 (19.6)	13 (16.0)	17 (23.6)	0.436

^TPP was performed at the 3 Indian centers and SPP at the French center.

*10/80 (12.5%) patients in whom BRCA testing was done.

**Table 3:** Major complications occurring within 90-days of surgery in patients undergoing interval cytoreductive surgery with or without HIPEC.

Complication	All patients n = 153 (%)	No HIPEC N = 55 (%)	HIPEC N = 98 (%)	p-value
Total number of patients with major complications	45 (29.4)	9 (16.3)	36 (36.7)	0.002
Haemorrhage	6 (3.9)	1 (1.8)	5 (5.1)	0.315
Bowel fistula	6 (3.9)	3 (5.4)	3 (3.0)	0.507
Intestinal perforation	0 (0.0)	0 (0.0)	0 (0.0)	.
Anastomotic leak	0 (0.0)	0 (0.0)	0 (0.0)	.
Other GI complications	6 (3.9)	1 (1.8)	5 (5.1)	0.097
Respiratory complications	9 (5.8)	4 (7.2)	5 (5.1)	0.583
Cardiac complications	7 (4.5)	1(1.8)	6 (6.1)	0.221
Urologic complications	0 (0.0)	0 (0.0)	0 (0.0)	.
Nephrotoxicity	2 (1.3)	0 (0.0)	2 (2.0)	0.924
Hematologic toxicity	15 (9.8)	0 (0.0)	15 (15.3)	0.052
Neutropenia	0 (0.0)	0 (0.0)	0 (0.0)	.
Systemic sepsis	3 (1.9)	1 (1.8)	2 (2.0)	0.924
Surgical site infection	3 (1.9)	0 (0.0)	3 (3.0)	0.650
Wound dehiscence	2 (1.3)	2 (3.6)	0 (0.0)	0.262
Intrabdominal abscess	1 (0.6)	1 (1.8)	0 (0.0)	0.656
Post op ascites/fluid collection	9 (5.8)	5 (9.0)	4 (4.0)	0.206
90-day post-operative mortality	3 (1.9)	0 (0.0)	3 (3.0)	0.650

### Pathological findings

The median pathological PCI was 8[range, 0-26] **(** **Table 1** **).** A complete pathological response to NACT was observed in 4 (2.6%) patients and a near complete response in 23 (15.0%). Regional lymph nodes were involved in 46(30.0%) patients. There was residual disease in the upper regions in 94(61.4%) patients and in the small bowel mesentery in 55(35.9%) on pathological evaluation.

### Early recurrence

**Figure 1:**
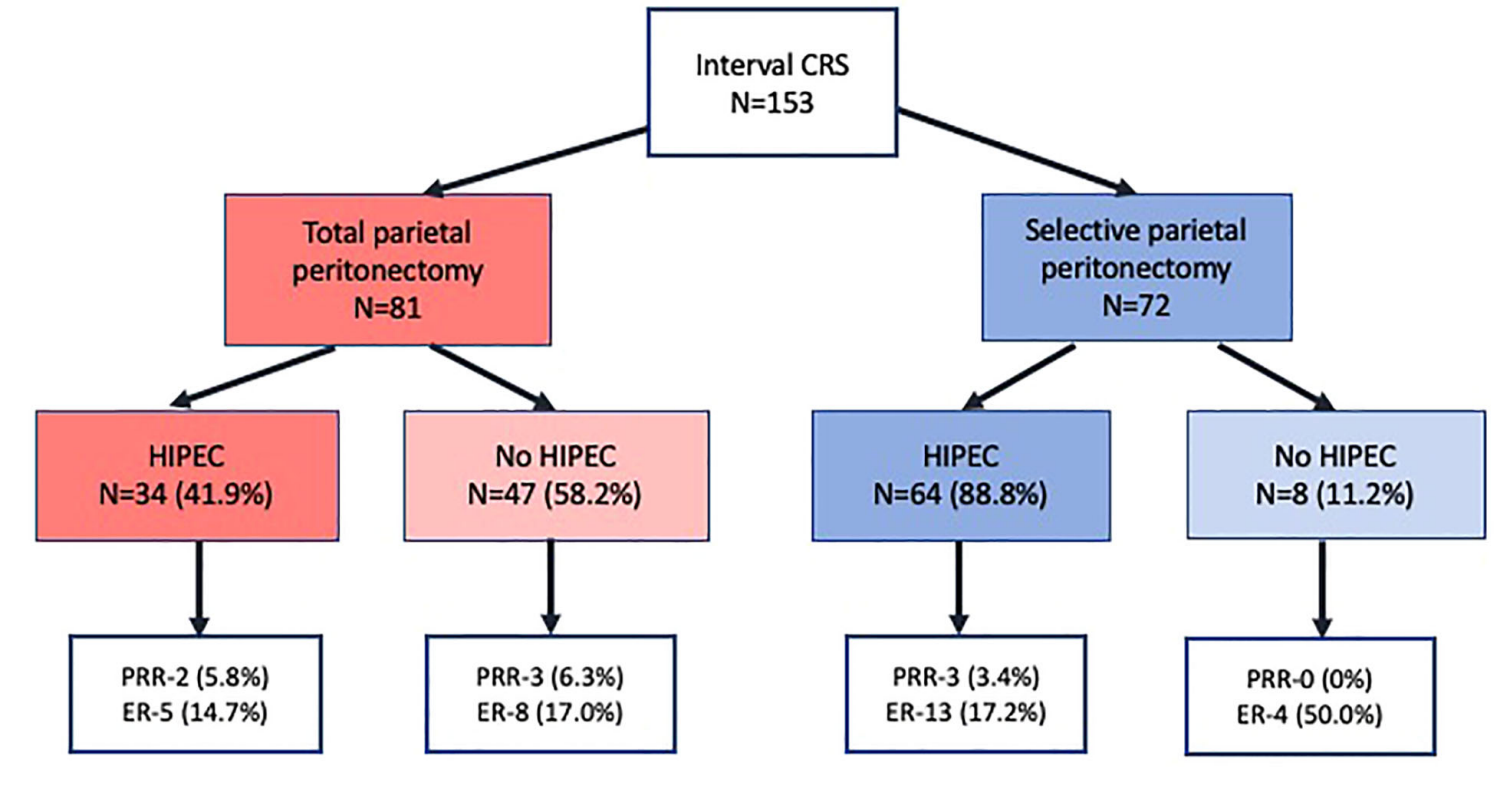
Platinum resistant recurrence and early recurrence in 153 patients undergoing interval cytoreductive surgery.

At a median follow-up of 16 months (range, 0-33 months), 46(30.0%) patients developed recurrence or disease progression. Of these, 10(6.5%) patients died of progressive disease. Platinum resistant recurrence (PRR) was observed in 8(5.2%) patients and recurrence within 6-12 months in 22(14.3%). Thus, 30(19.6%) patients developed early recurrence/disease progression (ER). Overall, 134 (87.5%) patients had completed 12 months of follow-up and in these, ER was seen in 23(17.1%) of these 134 patients. The ER of 17.1% in patients with 12 months of follow-up was lower than that of the whole cohort (19.6%) as patients with recurrence within 6-12 months who had not completed 12 months were excluded. Of the 19(12.5%) patients who did not have 12 months of follow-up, 3(1.9%) were dead due to postoperative complications and 4(2.6%) had died of progressive disease. The incidence of PRR and ER in different subgroups in shown in **Figure 1**.

### Factors affecting early recurrence (ER)

On multivariate logistic regression analysis, CC-0 (p=0.014) resection and HIPEC (p=0.030) were associated with reduced recurrence within 12 months **(** **Table 4** **)**. This analysis was performed only on the 134 patients that had a 12-month follow-up. A comparison of PRR and ER observed in this study with published literature is provided in **Table 5**. Though 25% of the patients had a PCI>20 and 75% had a PCI>10, PCI had no impact on the ER (only the comparison between PCI<20 and >20 is presented in this manuscript). Similarly, though a chemotherapy response grade of 3 was significant in the univariate analysis, it was not an independent predictor of ER. Due to the small number of patients, the factors affecting platinum resistant recurrence could not be evaluated.

**Table 4:** Factors affecting recurrence within 12 months of surgery (logistic regression analysis)*.

Prognostic variable (N)		Univariate analysis	Multivariate analysis	
		p-value	Hazard ratio [95% CI]	p-value
Surgical PCI	<20 >20	0.510		
CC-score	CC-0 CC-1-3	0.001	2.98 [2.5-38]	0.014
Pathological PCI	<15 >15	0.331		
HIPEC	Yes No	0.121		
Grade 3-4 complications	Yes No	0.121		
Lymph node involvement	Yes No	0.490		
Chemotherapy response grade	3** 1-2	0.031	NS^	
Extent of peritoneal resection	TPP SPP	0.570		
HIPEC	Yes No	0.020	1.77 [1.1-20]	0.030
Use of maintenance bevacizumab	Yes No	0.590		

*This analysis was performed on 134 patients who had completed 12 months of follow-up.

**Includes patients with a complete and near complete response.

^NS, Not significant.

**Table 5:** Platinum resistant recurrence and early recurrence observed in the current study and that reported in published literature.

Sub-group [ref]	N	CC0/1	Optimal debulking	Platinum resistant recurrence N (%)	Early recurrence N(%)
Current study All patients	153	96.6%	100%	8 (5.2)	30 (19.6) 23(17.1%)/134 with 12 months follow-up
SOLO-1 trial Interval CRS (Olaparib arm) (24)	94	81%	–	12 (12.7)	23 (24.4)
SOLO-1 trial CC-0 resection (Olaparib arm) (24)	200	100%	–	23 (11.5)	33(16.5)
SOLO-1 trial -BRCA mutations (Olaparib arm) (24)	257	76.6%	–	31(12.0)	56 (21.7)
PRIMA trial (Niriparib arim) (14)	487	–	–	175 (35.9)	320 (65.7)
PAOLA-1 -BRCA mutated tumors (olaparib arm)	157	–	–	7 (3.8)	13 (8.2)
EORTC-NCIC trial NACT arm (25)	334	45.5%	80.6%	–	179 (53.5)
CHORUS trial NACT arm (26)	274	39%	73%	76 (27.7)	155 (56.5)
SCORPION trial (NACT arm) (27)	87	77%	98.6%	–	24(27.5)
OVIHIPEC-1 trial; HIPEC arm (10)	122	69%	98%	–	55 (45.0)

### Patients treated with or without HIPEC

There were more CC-2/3 resections in patients not undergoing HIPEC (p=0.007) **(** **Table 1** **)**. The proportion of patients undergoing SPP (p<0.001) and receiving maintenance bevacizumab (p<0.001) was higher in the HIPEC group. Major complications (including the systemic toxicity caused due to HIPEC) were significantly higher in the HIPEC group (**Table 3** **)**. Platinum resistant recurrence(p=0.107) as well as early recurrence (p=0.972) were similar in the two groups.

### Patients treated with TPP or SPP

Patients treated with TPP were younger (p=0.009) and this group had more patients with a surgical PCI>10 (p=0.060)**(** **Table 2** **)**. The number of peritonectomies (p<0.001) and visceral resections (p=0.004) was higher in the TPP group. More patients undergoing SPP were treated with HIPEC (p<0.001) and maintenance bevacizumab (p<0.001). The incidence of platinum resistant recurrence (4.9% versus 4.1%; p=0.577) and early recurrence (16.0% versus 23.6%; p=0.436) was similar in the two groups. However, recurrence within 6-12 months was higher in the SPP group (6.1%versus 19.4%; p=0.092) though this difference was not statistically significant.

### Patients treated with and without bevacizumab

The 31 patients who received maintenance therapy with bevacizumab were all treated with SPP and 29 of these patients were treated with HIPEC. Further details have been provided in **Table 6**.

**Table 6:** Comparison between patients treated with and without bevacizumab.

Clinical parameter		All patients N = 153 (%)	With Bev N = 31 (%)	Without Bev N = 122 (%)	p-value
Age	<50 >50	28 (18.3) 125 (81.7)	1 (3.2) 30 (96.8)	27 (22.1) 95 (77.9)	0.015
Number of NACT cycles	3-4 >4	101 (66.0) 52 (34.0)	20 (64.5) 11(35.5)	81 (66.3) 41 (33.7)	0.843
Surgical PCI	0-9 10-19 20-29 30-39	37 (24.1) 77 (50.3) 36 (23.5) 3 (1.9)	9 (29.0) 18 (58.0) 4 (12.9) 0 (0.0)	28 (22.9) 59 (48.3) 32 (26.2) 3 (2.4)	0.442
Median surgical PCI		15 [0-37]	13 [3-28]	14 [0-31]	0.110
CC-score	CC-0 CC-1 CC-2/3	119 (77.7) 29 (18.9) 5 (3.2)	25 (80.6) 6 (19.4) 0 (0.0)	94 (77.0) 23 (18.8) 5 (4.0)	0.967
HIPEC		98	29 (93.5)	69 (56.5)	<0.001
Peritonectomy approach	TPP SPP	81(53.0) 72 (47.0)	0 (0.0) 31 (100.0)	81(66.3) 41 (33.7)	<0.001
Number of peritonectomies	0-6 7	54 (35.2) 99 (64.8)	19 (61.2) 12 (38.8)	35 (28.6) 87 (71.4)	<0.001
Organ resections	0-3 4-5 >5	71(46.4) 59 (38.5) 23 (15.0)	18 (58.0) 11(35.5) 2 (6.4)	53 (43.4) 48 (39.3) 21 (17.2)	0.208
Grade 3-4 complications		45 (29.4)	5 (6.1)	40 (32.7)	0.069
90-day mortality		3 (1.9)	0 (0.0)	3(2.4)	0.811
Pathological PCI	0-9 10-19 20-29 30-39	85 (55.5) 61 (39.8) 7 (5.5) 0 (0.0)	17 (54.8) 12 (38.8) 2 (6.4) 0 (0.0)	68 (55.7) 49 (40.1) 5 (4.0) 0 (0.0)	0.853
Median pathological PCI		8 [0-26]	7 [0-21]	9 [0-26]	0.540
Involvement of upper regions		94 (61.4)	19 (61.2)	75 (61.4)	0.984
Small bowel involvement		55 (35.9)	13 (41.9)	42 (34.4)	0.436
Chemotherapy response score		4 (2.6) 23 (15.0) 126 (82.3)	0 (0.0) 5 (16.1) 26 (83.9)	4 (3.2) 18 (14.7) 100 (81.9)	0.991
Regional lymph node involvement		46 (30.0)	9 (29.0)	36 (29.5)	0.958
Recurrence in 0-6 months of surgery		8 (5.2)	1 (3.2)	7 (6.5)	0.574
Recurrence in 6-12 months of surgery		22 (14.3)	7 (22.5)	15 (12.2)	0.144
Recurrence in -12 months		30 (19.6)	8 (36.3)	22 (18.0)	0.330

### Site of recurrence

Due to the retrospective nature of this study, the description of sites of recurrence differed among different centers. At one center, they were reported as peritoneal and extra-peritoneal (included nodal and visceral metastases) whereas the other centers recorded every site of disease recurrence. All eight patients with PRR had peritoneal involvement of which half the patients had isolated peritoneal recurrence **(** **Table 7** **)**. Of the 30% that developed ER, 40% had isolated peritoneal recurrence, 30% had only extra-peritoneal recurrence while 30% had peritoneal and extraperitoneal recurrence both **(** **Table 8** **)**. There was no significant difference in the peritoneal and non-peritoneal recurrence between patients undergoing TPP and SPP and those receiving and not-receiving HIPEC **(** **Table 9** **)**.

**Table 7:** Sites of recurrence in 8 patients who developed platinum resistant recurrence.

	Peritoneum alone	Peritoneal and extraperitoneal	Peritoneal and pleural	Peritoneal and nodal
All patients (n = 8)	4	2	1	1
TPP (N = 5)	2	1	1	1
SPP (N = 3)	2	1	0	0
HIPEC (N = 5)	3	1	0	1
No HIPEC (N = 3)	1	1	1	0

TPP, total parietal peritonectomy; SPP, selective parietal peritonectomy; HIPEC, hyperthermic intraperitoneal chemotherapy.

**Table 8:** Sites of recurrence in all 30 patients who developed early recurrence.

Treatment group	Peritoneum alone N (%)	Peritoneal and extraperitoneal N (%)	Extraperitoneal alone N (%)	Nodal alone* N (%)	Peritoneal and pleural* N (%)	Peritoneal and nodal* N (%)	Liver alone* N (%)
All patients (N = 30)	12 (40)	4 (13.3)	7 (23.3)	1(3.3)	2 (6.6)	3 (10)	1(3.3)
TPP (N = 13)	5 (38.4)	0 (0.0)	1 (7.6)	1 (7.6)	2 (15.3)	3(23.0)	1(7.6)
SPP (N = 17)	7 (41.1)	4 (23.5)	6 (35.2)	0 (0.0)	0 (0.0)	0 (0.0)	0 (0.0)
HIPEC (N = 18)	7 (38.8)	3 (16.6)	6 (33.3)	0 (0.0)	0 (0.0)	1(5.5)	1(5.5)
No-HIPEC (N = 12)	5 (41.6)	2 (16.6)	1 (8.3)	1 (8.3)	2 (16.6)	1(8.3)	0 (0.0)

*Some of these recurrences were reported as extra-peritoneal or peritoneal and extra-peritoneal.

TPP, total parietal peritonectomy; SPP, selective parietal peritonectomy; HIPEC, hyperthermic intraperitoneal chemotherapy.

**Table 9:** Peritoneal and non-peritoneal recurrence in 8 patients that developed platinum resistant recurrence and 30 patients that developed early recurrence.

Treatment group	All sites of recurrence	Peritoneal recurrence	Non-peritoneal recurrence	p-value
Platinum Resistant Recurrence				
All patients (N = 153)	8 (5.2%)	8 (100%)	0 (0.0)	
TPP (N = 81)	5 (3.7%)	5 (100%)	0 (0.0)	0.673
SPP (N = 72)	3 (4.1%)	3 (100%)	0 (0.0)	
HIPEC (N = 98)	3 (3.0)	3 (100%)	0 (0.0)	0.709
No- HIPEC (N = 55)	1 (1.8)	1 (100%)	0 (0.0)	
Early Recurrence				
All patients (N = 153)	30 (19.6)	21 (70.0)	9 (30.0)	
TPP (N = 81)	13 (16.0)	10 (76.9)	3 (23.1)	0.469
SPP (N = 72)	17 (23.6)	11 (64.7)	6 (35.3)	
HIPEC (N = 98)	18 (18.3)	11 (61.1)	7 (38.9)	0.193
No- HIPEC (N = 55)	12 (21.8)	10 (83.3)	2 (16.7)	
PCI	15 [0-37]	18 [5-37]	10 [2-19]	0.213

TPP, total parietal peritonectomy; SPP, selective parietal peritonectomy; HIPEC, hyperthermic intraperitoneal chemotherapy; PCI, peritoneal cancer index.

## Discussion

In this study incidence of platinum resistant recurrence (5.2%) and early recurrence(19.6%) following the last dose of platinum based therapy was low compared to historical data from randomized trials that included patients undergoing interval CRS **(** **Table 5** **)**. HIPEC and CC-0 resection were the only independent predictors of a low ERR.

PRR is an important end-point in ovarian cancer as it is associated with a poorer response to subsequent chemotherapy and a poorer overall survival (28). Though patients with asymptomatic recurrence may have a better outcome than those with symptomatic recurrence, the overall prognosis of these patients is poorer compared to those with platinum sensitive disease (28). Similarly, patient who recur from 6-12 months have partially platinum sensitive disease that has a poorer outcome compared to platinum sensitive recurrence.

### The impact of aggressive/extensive surgery

There were two surgical strategies– resecting only sites of residual disease and resecting the entire parietal peritoneum along with viscera bearing residual disease. In this regard, only patients with stage III-C that have unresectable disease at presentation are included in the study and the entire parietal peritoneum is usually involved in these patients. Thus, in patients treated with TPP, peritoneum that was never involved is not removed. Previous studies on the distribution of residual disease have shown that following NACT, the parietal peritoneum is the most common site of occult disease (7). The visceral peritoneum (except the omenta) has less occult and overt disease both. Occult disease following NACT harbors chemotherapy resistant stem cells that may not be eradicated completely with adjuvant chemotherapy and TPP is performed to address this disease more effectively (4).

The SPP performed in this study was performed at an expert center where the entire peritoneal region in which the disease bearing peritoneum region lies is resected. This surgery is likely to be more extensive than the SPP performed at many other gynecologic oncology units considering that nearly 80% of patients had a diaphragmatic peritonectomy in the SPP group. Upper abdominal procedures were performed in 37.8% patients in the NACT arm of the SCORPION trial (29).The surgeons also systematically resected the lesser omentum, the falciform and umbilical round ligament that are common sites of residual disease. Another difference was the significantly higher number of patients receiving both HIPEC and bevacizumab in the SPP group. These could be some of the reasons for the lack of difference in PRR and ER between the TPP and SPP groups.

It has been clearly demonstrated that there is benefit of having no residual disease (complete gross resection-CGR) following NACT over optimal cytoreduction (<1cm residual disease) (3, 27). And it may be questioned why the benefit should stop at a CGR and not be obtained when the occult disease is resected more completely. Even with a TPP, it is impossible to identify and resect all sites of occult disease but the amount of occult residual disease can be substantially reduced.

### HIPEC

HIPEC has shown a benefit in PFS and OS both in addition to CRS alone in the interval setting (10). HIPEC addresses microscopic residual disease and the combination of cisplatin with heat has the potential to overcome platinum resistance (30). A significantly higher proportion of patients in the SPP group received HIPEC, which could be another factor responsible for the similar rates of PRR and ER in the two groups. Assumedly, HIPEC should add to the benefit of TPP and may not be replacement for it. Whereas a TPP removes occult disease from the parietal peritoneum more effectively, HIPEC has the additional benefit of addressing free intraperitoneal cancer cells shed during surgery and preventing their implantation at sites of resection. The benefit of the combination of TPP and HIPEC should be evaluated in future studies.

### Impact of other prognostic factors

Though PCI is not an established prognostic factor in advanced ovarian cancer, several studies have shown an inferior survival in patients with a high PCI (31–33). This factor had no impact on ER in this study. Thus, even patients with more extensive surgery (25% with PCI>20 in this study) had a low PRR and ER in this study.

Chemotherapy response grade was not an independent predictor of ER and it may be inferred that TPP and HIPEC could delay recurrence in sub-groups of patients that have a poorer response to systemic chemotherapy (22).

### Morbidity and mortality

The overall major morbidity of 30% and mortality of 1.9% compares well with published literature and could be considered acceptable (24, 25, 34). The 90-day morbidity was considered and even the systemic toxicity was included in this evaluation which explain the incidence of 30%. The morbidity was significantly higher in the HIPEC group **(** **Table 3** **)**. This was mainly due to the hematological side effect of HIPEC which are not observed in patients that do not undergo HIPEC.

The morbidity in the SPP group was also higher due to more number of patients receiving HIPEC in this group. There was no mortality in the SPP group and all patients started adjuvant chemotherapy within 6 weeks of surgery. Three deaths occurred in the TPP group and all three patients received HIPEC. This is the average rate of post-operative mortality at Indian centers as reported in previous studies (7, 26). One patient died of hemorrhagic shock and two others of systemic sepsis that occurred in absence of gastrointestinal complications.

### Maintenance therapy with bevacizumab

Maintenance therapy with bevacizumab has shown a benefit in overall-survival in patients with suboptimal debulking and those with stage IV disease (11, 12). In all the trials evaluating the role of maintenance bevacizumab, It’s benefit in patients who have a complete cytoreduction has not been demonstrated (11, 12). The use of bevacizumab was at the discretion of the treating physician in this study and in the univariate analysis it had no impact on ER. It has been shown that the benefit of bevacizumab is short lived and wears of soon after discontinuation of therapy. The optimal duration of maintenance therapy with bevacizumab has still not been determined. We presume that bevacizumab should be an adjunct to aggressive locoregional therapies and not a substitute for them and its role in patients undergoing TPP and/or HIPEC should be evaluated in future studies.

### Maintenance therapy with PARP inhibitors

Similarly, PARP inhibitors were not used for all patients, even those with BRCA mutations as the evidence for its benefit in different subgroups was only evolving at the time of this study. For Indian patients, the cost is the main limiting factor. In patients with BRCA 1 and 2 mutations in different randomized trial, the PRR and ER rates were similar or more than those in our study**(** **Table 5** **).** This comparison is not ideal considering that the intention-to-treat population is considered in the survival analysis in these trials and that includes approximately 10-15% of the patients that never had surgery. But even if these patients were excluded, the reduction in the PRR and ER would not be more than 2-3%. Thus, similar rates of ER and PRR were achieved with our locoregional strategies without the maintenance therapies. In the subgroup analysis of the SOLO 1 trial, 11.5% of the patients with a CGR recurred at 6 months and 16.7% at 12 months which is similar to the results in this study **(** **Table 5** **)** (35). The benefit of aggressive locoregional therapies in patients with BRCA mutations who receive maintenance therapy needs further evaluation; our presumption is that the benefit could be additive.

### Site of PRR and ER

Though the reporting of sites of recurrence was not uniform, we were able to distinguish between the peritoneal and non-peritoneal recurrences. All patients with PRR had peritoneal recurrences while 70% of the ERs were peritoneal with or without extra-peritoneal recurrences. There is limited information on the sites of recurrence in patients with PRR in literature. Petrillo et al. found peritoneal recurrence in nearly 50% and isolated nodal recurrence in the remaining 50% of the patients undergoing secondary CRS for PRR (36). They did not report the sites of recurrence in the whole cohort of 268 patients with PRR and hence our findings cannot be compared to this study. The incidence of isolated nodal recurrences in this study was low though we have not been able to capture the exact incidence. It has been shown that patients with isolated nodal recurrences are more likely to undergo secondary CRS and these recurrences are less chemosensitive (37). There was no difference in the peritoneal recurrence rate in patients undergoing TPP and SPP though this comparison is not ideal since a significantly higher number of patients in the SPP group received HIPEC. TPP and HIPEC should both reduce the incidence of peritoneal recurrence and thus, prolong survival. Our findings however cannot be generalized to all patients in this study as the sites of late recurrence may not be the same as that of early recurrence. Moreover, not all peritoneal recurrences are the same- there are isolated recurrences that are amenable to surgery, non-isolated asymptomatic recurrences and more widespread recurrences that produce symptoms early on. The pattern of recurrence following TPP should be an area of future study.

### Strengths, limitations and future directives

This study has many limitation beginning with the inherent bias that exists in all retrospective studies. The number of patients in different subgroups is small (TPP versus SPP and HIPEC versus no-HIPEC). The major shortcoming of this study is the comparison of different populations: the SPP patients were French, and routinely underwent HIPEC after CRS. In the Indian population, which is fundamentally different in terms of the healthcare system, HIPEC was only performed in patients who can afford the cost of treatment. The use of maintenance therapies was not uniform which adds to the heterogeneity in the patient population. The main strengths of this study are that data were collected prospectively and surgery was performed according to predefined protocols at all centers. Meticulous disease mapping was done during surgery and on pathology using the PCI. The study included patients with extensive disease- over 60% had residual disease in the upper abdominal regions and 35% on the small bowel mesentery on pathology. Despite the limitations of this study, the reduction in both PRR and ER is significant (75%) compared to that reported in randomized trials on interval CRS which is the main reason for presenting these results early on **(** **Table 5** **)** (38, 39). These results need to be confirmed in larger and more homogeneous patient cohorts. The follow-up is short but is adequate to evaluate the incidence of PRR and 87.5% had completed 1 year of follow-up which is sufficient to evaluate ER. Both PRR and ER are important end-points in ovarian cancer as delaying recurrence is essential associated with a longer platinum-free interval that is a robust prognostic factor in advanced ovarian cancer (28). The benefit of aggressive locoregional therapies is that they are ‘single-shot’ treatments and can provide a longer ‘treatment-free’ and ‘platinum-free’ interval compared to conventional surgery but the role of these treatments in the light of maintenance therapies needs further evaluation. For TPP, the impact on PFS and OS has still not be demonstrated. This study is retrospective and the results are applied to generate new hypotheses and we do not recommend any practice changes based on these results.

## Conclusions

The incidence of PRR and ER in this cohort was low compared to historical data. HIPEC and CC-0 resection were independent predictors of a low ER. These results should be confirmed in larger and more homogeneous patient cohorts. Future research should evaluate the potential additive benefit of aggressive locoregional therapies like TPP and HIPEC coupled together as well as their combination with maintenance therapies.

## Data availability statement

The raw data supporting the conclusions of this article will be made available by the authors, without undue reservation.

## Ethics statement

The studies involving human participants were reviewed and approved by Zydus Hospital Ethics Committee, India. The patients/participants provided their written informed consent to participate in this study.

## Author contributions

All the authors have made a substantial contribution to this manuscript as described below. Study concept: AB, OG, VK, and SSi. Study design: AB, OG, VK, and SSi. Data collection: AB, SSi, SSh, PK, WG, VK, OG, NB, and SM. Data analysis and interpretation: AB and SSi. Statistical analysis: SSi and AB. Manuscript preparation: AB, VK, and OG. Manuscript editing: All authors. Manuscript review: All authors. The final version of the manuscript was approved by all the authors. All the authors agree are accountable for all aspects of the work in ensuring that questions related to the accuracy or integrity of any part of the work are appropriately investigated and resolved.

## Conflict of interest

OG is a consultant for Gamida Tech.

The remaining authors declare that the research was conducted in the absence of any commercial or financial relationships that could be construed as a potential conflict of interest.

## Publisher’s note

All claims expressed in this article are solely those of the authors and do not necessarily represent those of their affiliated organizations, or those of the publisher, the editors and the reviewers. Any product that may be evaluated in this article, or claim that may be made by its manufacturer, is not guaranteed or endorsed by the publisher.

## References

[1] Reuss A du Bois A Harter P Fotopoulou C Sehouli J Aletti G. TRUST: Trial of radical upfront surgical therapy in advanced ovarian cancer (ENGOT ov33/AGO-OVAR OP7). Int J Gynecol Cancer (2019) 29(8):1327–31. doi: 10.1136/ijgc-2019-000682 31420412

[2] Liu J Jiao X Gao Q. Neoadjuvant chemotherapy-related platinum resistance in ovarian cancer. Drug Discov Today (2020) 25(7):1232–38. doi: 10.1016/j.drudis.2020.04.015 32360532

[3] Bristow RE Chi DS. Platinum-based neoadjuvant chemotherapy and interval surgical cytoreduction for advanced ovarian cancer: A meta-analysis. Gynecol Oncol. (2006) 103(3):1070–6. doi: 10.1016/j.ygyno.2006.06.025 16875720

[4] Lim MC Song YJ Seo SS Yoo CW Kang S Park SY. Residual cancer stem cells after interval cytoreductive surgery following neoadjuvant chemotherapy could result in poor treatment outcomes for ovarian cancer. Onkologie (2010) 33(6):324–30. doi: 10.1159/000313823 20523098

[5] Bhatt A Kammar P Mehta S Sinukumar S. ASO author reflections: Total parietal peritonectomy during interval cytoreductive surgery for advanced ovarian cancer–Proof-of-Principle and analysis of morbidity. Ann Surg Oncol (2020) 27(Suppl 3):861–2. doi: 10.1245/s10434-020-08940-6 32720047

[6] Sinukumar S Rajan F Mehta S Damodaran D Zaveri S Kammar P. A comparison of outcomes following total and selective peritonectomy performed at the time of interval cytoreductive surgery for advanced serous epithelial ovarian, fallopian tube and primary peritoneal cancer - a study by INDEPSO. Eur J Surg Oncol (2019) 47(1):75–81. doi: 10.1016/j.ejso.2019.02.031 30857879

[7] Bhatt A Kammar P Sinukumar S Parikh L Jumle N Shaikh S. Total parietal peritonectomy can be performed with acceptable morbidity for patients with advanced ovarian cancer after neoadjuvant chemotherapy: Results from a prospective multi-centric study. Ann Surg Oncol (2021) 28(2):1118–29. doi: 10.1245/s10434-020-08918-4 32748154

[8] Bhatt A Sinukumar S Mehta S Damodaran D Zaveri S Kammar P. Patterns of pathological response to neoadjuvant chemotherapy and its clinical implications in patients undergoing interval cytoreductive surgery for advanced serous epithelial ovarian cancer- a study by the Indian network for development of peritoneal surface oncology (INDEPSO). Eur J Surg Oncol (2019) 45(4):666–71. doi: 10.1016/j.ejso.2019.01.009 30661922

[9] Bhatt A Bakrin N Kammar P Mehta S Sinukumar S Parikh L. Distribution of residual disease in the peritoneum following neoadjuvant chemotherapy in advanced epithelial ovarian cancer and its potential therapeutic implications. Eur J Surg Oncol (2021) 47(1):181–7. doi: 10.1016/j.ejso.2020.10.012 33071172

[10] van Driel WJ Koole SN Sikorska K Schagen van Leeuwen JH Schreuder HWR Hermans RHM. Hyperthermic intraperitoneal chemotherapy in ovarian cancer. N Engl J Med (2018) 378:230–40. doi: 10.1056/NEJMoa1708618 29342393

[11] Perren TJ Swart AM Pfisterer J Ledermann JA Pujade-Lauraine E Kristensen G. A phase 3 trial of bevacizumab in ovarian cancer. N Engl J Med (2011) 365:2484–96. doi: 10.1056/NEJMoa1103799 22204725

[12] Tewari KS Burger RA Enserro D Norquist BM Swisher EM Brady MF. Final overall survival of a randomized trial of bevacizumab for primary treatment of ovarian cancer. J Clin Oncol (2019) 37(26):2317–28. doi: 10.1200/JCO.19.01009 PMC6879307 31216226

[13] Moore K Colombo N Scambia G Kim BG Oaknin A Friedlander M. Maintenance olaparib in patients with newly diagnosed advanced ovarian cancer. N Engl J Med (2018) 379:2495–505. doi: 10.1056/NEJMoa1810858 30345884

[14] Gonzalez-Martin A Pothuri B Vergote I DePont Christensen R Graybill W Mirza MR. Niraparib in patients with newly diagnosed advanced ovarian cancer. N Engl J Med (2019) 381:2391–402. doi: 10.1056/NEJMoa1910962 31562799

[15] Jacquet P Sugarbaker PH. Clinical research methodologies in diagnosis and staging of patients with peritoneal carcinomatosis. Cancer Treat Res (1996) 82:359–74. doi: 10.1007/978-1-4613-1247-5_23 8849962

[16] Bhatt A Yonemura Y Mehta S Benzerdjeb N Kammar P Parikh L. Target region resection in patients undergoing cytoreductive surgery for peritoneal metastases-is it necessary in absence of visible disease? Eur J Surg Oncol J Eur Soc Surg Oncol Br Assoc Surg Oncol (2020) 46(4 Pt A):582–9. doi: 10.1016/j.ejso.2019.11.49 31757660

[17] United States department of public health and human services, NIH, NCI. In: Common toxicity criteria for adverse events (CTCAE). USA National Cancer Institute. Available at: https://evs.nci.nih.gov/ftp1/CTCAE/CTCAE_4.03/CTCAE_4.03_2010-06-14_QuickReference_8.5x11.pdf.

[18] Bhatt A Yonemura Y Benzerdjeb N Mehta S Mishra S Parikh L. Pathological assessment of cytoreductive surgery specimens and its unexplored prognostic potential- a prospective multi-centric study. Eur J Surg Oncol (2019) 45(12):2398–404. doi: 10.1016/j.ejso.2019.07.019 31337527

[19] McCluggage WG Judge MJ Clarke BA Davidson B Gilks CB Hollema H. Data set for reporting of ovary, fallopian tube and primary peritoneal carcinoma: recommendations from the international collaboration on cancer reporting (ICCR). Mod Pathol (2015) 28(8):1101–22. doi: 10.1038/modpathol.2015.77 26089092

[20] Villeneuve L Thivolet A Bakrin N Mohamed F Isaac S Valette PJ. A new internet tool to report peritoneal malignancy extent. PeRitOneal MalIgnancy stage evaluation (PROMISE) application. Eur J Surg Oncol (2016) 42(6):877–82. doi: 10.1016/j.ejso.2016.03.015 27067193

[21] Bhatt A Yonemura Y Mehta S Benzerdjeb N Kammar P Parikh L. The pathologic peritoneal cancer index (PCI) strongly differs from the surgical PCI in peritoneal metastases arising from various primary tumors. Ann Surg Oncol (2020) 27(8):2985–96. doi: 10.1245/s10434-020-08234-x 32040698

[22] Böhm S Faruqi A Said I Lockley M Brockbank E Jeyarajah A. Chemotherapy response score: development and val- idation of a system to quantify histopathologic response to neoad- juvant chemotherapy in tubo-ovarian high-grade serous carcino- ma. J Clin Oncol (2015) 33(22):2457–63. doi: 10.1200/JCO.2014.60.5212 26124480

[23] Rustin GJ Vergote I Eisenhauer E Pujade-Lauraine E Quinn M Thigpen T. Definitions for response and progression in ovarian cancer clinical trials incorporating RECIST 1.1 and CA 125 agreed by the gynecological cancer intergroup (GCIG). Int J Gynecol Cancer (2011) 21:419–23. doi: 10.1097/IGC.0b013e3182070f17 21270624

[24] Di Giorgio A De Iaco P De Simone M Garofalo A Scambia G Pinna AD. Cytoreduction (peritonectomy procedures) combined with hyperthermic intraperitoneal chemotherapy (HIPEC) in advanced ovarian cancer: retrospective Italian multicenter observational study of 511 cases. Ann Surg Oncol (2017) 24(4):914–22. doi: 10.1245/s10434-016-5686-1 PMC5339330 27896512

[25] Alyami M Kim BJ Villeneuve L Vaudoyer D Képénékian V Bakrin N. Ninety-day post-operative morbidity and mortality using the national cancer institute’s common terminology criteria for adverse events better describe post-operative outcome after cytoreductive surgery and hyperthermic intraperitoneal chemotherapy. Int J Hyperth (2018) 34(5):532–7. doi: 10.1080/02656736.2017.1367846 28838265

[26] Sinukumar S Mehta S Damodaran D Rajan F Zaveri S Ray M. Failure-to-Rescue following cytoreductive surgery with or without HIPEC is determined by the type of complication-a retrospective study by INDEPSO. Indian J Surg Oncol (2019) 10(Suppl 1):71–9. doi: 10.1007/s13193-019-00877-x PMC6397122 30886497

[27] Ghirardi V Moruzzi MC Bizzarri N Vargiu V D'Indinosante M Garganese G. Minimal residual disease at primary debulking surgery versus complete tumor resection at interval debulking surgery in advanced epithelial ovarian cancer: A survival analysis. Gynecol Oncol (2020) 157(1):209–13. doi: 10.1016/j.ygyno.2020.01.010 31952843

[28] Davis A Tinker AV Friedlander M. "Platinum resistant" ovarian cancer: what is it, who to treat and how to measure benefit? Gynecol Oncol (2014) 133(3):624–31. doi: 10.1016/j.ygyno.2014.02.038 24607285

[29] Fagotti A Ferrandina MG Vizzielli G Pasciuto T Fanfani F Gallotta V. Randomized trial of primary debulking surgery versus neoadjuvant chemotherapy for advanced epithelial ovarian cancer (SCORPION- NCT01461850). Int J Gynecologic Cancer (2020) 30(11):1657–64. doi: 10.1136/ijgc-2020-001640 33028623

[30] Hettinga JV Konings AW Kampinga HH. Reduction of cellular cisplatin resistance by hyperthermia — a review. Int J Hyperth (1997) 13:439–57. doi: 10.3109/02656739709023545 9354931

[31] Le Saux O Decullier E Freyer G Glehen O Bakrin N. Long-term survival in patients with epithelial ovarian cancer following cytoreductive surgery and hyperthermic intraperitoneal chemotherapy (HIPEC). Int J Hyperthermia (2018) 35(1):652–7. doi: 10.1080/02656736.2018.1518544 30295114

[32] Roosen A Sanson C Faron M Maulard A Pautier P Leary A. 1015 Linear relationship of peritoneal cancer index and survival in patients with epithelial ovarian cancer in carcinomatosis. Int J Gynecologic Cancer (2021) 31:A291–2.

[33] Elzarkaa AA Shaalan W Elemam D Mansour H Melis M Malik E. Peritoneal cancer index as a predictor of survival in advanced stage serous epithelial ovarian cancer: a prospective study. J Gynecol Oncol (2018) 29(4):e47. doi: 10.3802/jgo.2018.29.e47 29770618 PMC5981099

[34] Bakrin N Bereder JM Decullier E Classe JM Msika S Lorimier G. Peritoneal carcinomatosis treated with cytoreductive surgery and hyperthermic intraperitoneal chemotherapy (HIPEC) for advanced ovarian carcinoma: a French multicentre retrospective cohort study of 566 patients. Eur J Surg Oncol (2013) 39:1435–43. doi: 10.1016/j.ejso.2013.09.030 24209430

[35] DiSilvestro P Colombo N Scambia G Kim BG Oaknin A Friedlander M. Efficacy of maintenance olaparib for patients with newly diagnosed advanced ovarian cancer with a BRCA mutation: Subgroup analysis findings from the SOLO1 trial. J Clin Oncol (2020) 38(30):3528–37. doi: 10.1200/JCO.20.00799 PMC8190876 32749942

[36] Petrillo M Pedone Anchora L Tortorella L Fanfani F Gallotta V Pacciani M. Secondary cytoreductive surgery in patients with isolated platinum-resistant recurrent ovarian cancer: a retrospective analysis. Gynecol Oncol (2014) 134(2):257–61. doi: 10.1016/j.ygyno.2014.05.029 24910451

[37] Gallotta V Bruno M Conte C Giudice MT Davià F Moro F. Salvage lymphadenectomy in recurrent ovarian cancer patients: Analysis of clinical outcome and BRCA1/2 gene mutational status. Eur J Surg Oncol (2020) 46(7):1327–33. doi: 10.1016/j.ejso.2020.01.035 32085925

[38] Vergote I Trope CG Amant F Kristensen GB Ehlen T Johnson N. European Organization for research and treatment of cancer-gynaecological cancer group; NCIC clinical trials group. neoadjuvant chemotherapy or primary surgery in stage IIIC or IV ovarian cancer. N Engl J Med (2010) 363:943–53. doi: 10.1056/NEJMoa0908806 20818904

[39] Kehoe S Hook J Nankivell M Jayson GC Kitchener H Lopes T. Primary chemotherapy versus primary surgery for newly diagnosed advanced ovarian cancer (CHORUS): an open-label, randomised, controlled, non-inferiority trial. Lancet (2015) 386(9990):249–57. doi: 10.1016/S0140-6736(14)62223-6 26002111

